# Risk of Incident Immune-Mediated Inflammatory Diseases with Second Tumor Necrosis Factor Inhibitor Versus Alternative Biologic Therapy in Patients with Inflammatory Bowel Disease and First TNFi Exposure: A Real-World Cohort Study

**DOI:** 10.1007/s10620-025-09598-4

**Published:** 2025-12-09

**Authors:** Aakash Desai, Gursimran S. Kochhar, Himsikhar Khataniar, Jana G. Hashash, Francis A. Farraye

**Affiliations:** 1 Division of Gastroenterology, Hepatology & Nutrition, Allegheny Health Network, Pittsburgh, PA USA; 2 Drexel University College of Medicine, Philadelphia, PA USA; 3 Department of Medicine, Allegheny Health Network, Pittsburgh, PA USA; 4 Division of Gastroenterology and Hepatology, Mayo Clinic, Jacksonville, FL USA; 5 Division of Gastroenterology, Hepatology and Nutrition, Allegheny Health Network, 1307 Federal St., Suite 305, Pittsburgh, PA 15212 USA

**Keywords:** Crohn’s disease, Ulcerative colitis, Tumor necrosis factor inhibitors, Ustekinumab, Vedolizumab, Immune-mediated inflammatory diseases

## Abstract

**Introduction:**

Immune-mediated inflammatory diseases (IMIDs) can develop during tumor-necrosis-factor inhibitor (TNFi) therapy for inflammatory bowel disease (IBD). The impact of exposure to a second TNFi compared to alternative biologic therapy on the risk of IMIDs is unknown.

**Methods:**

Using the US Collaborative Network (2014–2023), we identified adults with Crohn’s disease (CD) or ulcerative colitis (UC) with previous exposure to a TNFi who were either switched to a second TNFi or ustekinumab/vedolizumab. Patients with any pre-existing IMID prior to switching to a second biologic were excluded. The primary outcome was the risk of IMID in the TNFi cohort compared to ustekinumab/vedolizumab cohort (reference treatment cohort) within 5 years. A 1:1 propensity score matching (PSM) was performed. Cox proportional hazard model was used to identify risk factors for new onset IMID in the TNFi cohort.

**Results:**

Among 14,360 patients, 5962 (41.5%) received a second TNFi (mean age 34.4 ± 16.7, 49.6% female, 73.9% White, 80% CD) and 8398 (58.5%) were switched to ustekinumab or vedolizumab (mean age 39.7 ± 17 years, 51.3% female, 75.3% White, 77.3% CD). After PSM, the second TNFi cohort had a higher risk of IMID compared to the reference treatment cohort (10.8% vs 6.9%, adjusted hazard ratio [aHR] 1.57, 95% CI 1.37–1.79). The increased risk was seen in both UC (aHR 1.90, 95% CI 1.53–2.37) and CD (aHR 1.43, 95% CI 1.22–1.67). Sensitivity analysis after excluding psoriasis, rheumatoid arthritis and ankylosing spondylitis also showed an increased risk of IMID in the TNFi cohort (aHR 1.67, 95% CI 1.37–2.05). Sub-group analysis based on age and sex also showed an increased risk of IMID in the TNFi cohort. Within the TNFi cohort, age ≥ 40 years, primary sclerosing cholangitis and methotrexate use predicted IMID, whereas male sex and concomitant azathioprine were protective.

**Discussion:**

In this large real-world IBD cohort with exposure to a TNFi, second TNFi use was associated with a higher risk of de-novo IMIDs compared to ustekinumab or vedolizumab.

**Supplementary Information:**

The online version contains supplementary material available at 10.1007/s10620-025-09598-4.

## Introduction

Inflammatory bowel disease (IBD) affects more than 7 million people worldwide, driving demand for durable and safe therapies [1]. Tumor-necrosis-factor inhibitors (TNFi) were the first advanced therapies to be approved for management of Crohn’s disease (CD) and ulcerative colitis (UC), and remain one of the most prescribed class of medications [2]. However, up to 40% of patients show primary non-response and up to 30% lose response annually [3, 4]. Immunogenicity is the predominant mechanism of secondary loss of response. In the prospective UK PANTS cohort (*n* = 1240), anti-drug antibodies (ADA) developed in 26% of infliximab- and 28% of adalimumab-treated patients within 12 months; ADA accounted for 38% of all treatment failures and led to permanent drug withdrawal in 14% [5]. Safety considerations are another common reason for stopping anti-TNF therapy: in the long-running TREAT registry, 9.8% of 6273 infliximab-exposed subjects discontinued because of serious infection or infusion reactions over a median five-year follow-up [6]. As a result, this requires clinicians to choose between switching within class to a second TNFi or changing to an advanced therapy with alternative mechanism like ustekinumab, which blocks interleukin-12/23, or vedolizumab, which inhibits α4β7-integrin–mediated gut homing [7]. Current algorithms compare effectiveness, pharmacokinetics, cost, and convenience, but long-term safety—particularly the emergence of new immune-mediated inflammatory diseases (IMIDs)—has become an equally important determinant.

Some agents that suppress an abnormal immune response in one context may provoke it in another [8]. Large nationwide cohorts from Denmark and France demonstrated a 76% increase in the composite risk of psoriasis, rheumatoid arthritis (RA), and hidradenitis suppurativa (HS) among TNFi-treated IBD patients compared with non-biologic controls [9], findings echoed in single-center series and meta-analyses [10]. Pathogenetically, prolonged neutralization of soluble TNF skews the cytokine milieu toward type I interferon and interleukin-17 production, activates plasmacytoid dendritic cells, and promotes autoreactive antibody formation—mechanisms thought to underlie paradoxical psoriasis and lupus-like syndromes [11]. Genetic susceptibility, epitope spreading after anti-drug antibody formation, and unmasking of subclinical autoimmunity have also been implicated [12]. In contrast, early registry data suggest ustekinumab and vedolizumab may confer a lower systemic IMID risk, but direct head-to-head comparisons after failure of a first TNFi are scarce. Only one French administrative study has addressed this question, reporting a 37% relative reduction in incident IMIDs after switching to ustekinumab versus a second TNFi, yet granular data on IBD phenotype, event timing, and patient-level modifiers were lacking [13].

Clarifying these uncertainties is clinically relevant because paradoxical IMIDs can produce morbidity, prompt biologic discontinuation, and necessitate additional immunosuppression. If within-class switching confers an excess autoimmune burden, choosing an alternative mechanism could maintain disease control while sparing patients systemic complications. Moreover, identifying subgroups at highest risk could personalize sequencing strategies and inform monitoring protocols. The primary aim of our study was to compare the incidence of IMIDs in adults with IBD who, after discontinuing a first TNFi, initiated either a second TNFi or switched class to ustekinumab or vedolizumab. The secondary aim was to assess the risk of IMIDs by age, sex, and IBD subtype while exploring independent predictors of IMID development among second TNFi users. By addressing this evidence gap, our study aims to guide shared decision-making and optimize the long-term safety of biologic therapy in IBD.

## Methods

### Database

A retrospective cohort study was conducted utilizing TriNetX (Cambridge, MA, USA), a multi-institutional database. TriNetX is a global federated research network which provides real-time access to de-identified electronic health records of more than 125 million patients within 106 health care organizations. Most health care organizations are large academic medical institutions which contain inpatient and outpatient facilities. The data represents the entire patient population of the organization. The de-identification process is determined and done at a network-level and attested to through a formal determination by a qualified expert as defined in the HIPAA Privacy Rule. TriNetX obfuscates patient counts < 10 to ensure patient anonymity. Clinical variables are derived directly from electronic health records of included health care organizations as well as retrieved through a built-in natural language processing system that extracts variables from clinical documents. Robust quality assurance is achieved at the time of extraction from electronic health records before inclusion in the database, in a systematic and standardized format. The process also includes data cleaning which rejects patient records that don’t meet the TriNetX quality standards. The database does not include claims data or data collected from randomized clinical trials. The database contains inpatient, and outpatient claims along with prescription drug claims. The interface only provides aggregate counts and statistical summaries to protect patient health information and ensures that the data remain de-identified at all levels of data retrieval and dissemination.

### Study Participants and Cohorts

We conducted a real-time search and analysis of the US Collaborative Network in the TriNetX database in adults with IBD who initiated either a second TNFi or ustekinumab/vedolizumab after previous exposure to a TNFi between January 1, 2010 and December 31, 2023. Patients with IBD were identified using International Classification of Disease, Tenth Revision, Clinical Modification (ICD-10-CM) codes in their EHR for Ulcerative colitis (K51*) or Crohn’s disease (K50*). All patients were required to initiate either infliximab or adalimumab as the first biologic therapy. Using the temporal relationship functionality, we divided the cohorts into patients who initiated a second TNFi or ustekinumab/vedolizumab (reference treatment cohort) following the initial TNFi prescription. Patients who had ICD-10-CM codes for any of the IMIDs of interest prior to initiation of second TNFi or ustekinumab/vedolizumab were excluded from the study.

### Study Outcomes

The primary outcome of the study was the 5-year risk of de-novo IMID following initiation of second biologic therapy. The index exposure was initiation of a second TNFi or ustekinumab/vedolizumab (reference treatment cohort). We used an intention-to-treat framework and did not define an on-treatment risk window or extend exposure by drug half-lives. Participants remained in their index cohort regardless of subsequent discontinuation of the index biologic or switching to another advanced therapy (e.g., risankizumab, upadacitinib). Follow-up began 30 days after the index date and ended at the earliest of incident IMID, 5 years, or last encounter in the network. IMIDs included in the study were psoriasis, rheumatoid arthritis (RA), ankylosing spondylitis (AS), systemic lupus erythematosus (SLE), vitiligo, hidradenitis suppurativa, polymyalgia rheumatica, sarcoidosis, Addison’s disease, Graves’ disease, autoimmune hepatitis (AIH), primary biliary cholangitis, pernicious anemia, multiple sclerosis, myasthenia gravis and idiopathic thrombocytopenic purpura. The individual risk of each IMID was also assessed. Sub-group analysis was performed based on age, sex and IBD type. Sensitivity analysis was conducted after exclusion of common IMID including psoriasis, rheumatoid arthritis and ankylosing spondylitis as well as following a 6-month washout. We also evaluated the risk of different co-variates on the risk of IMID in the second TNFi cohort. Co-variates included different age groups, male sex, race/ethnicity, nicotine dependence, primary sclerosing cholangitis, recent corticosteroid use, exposure to thiopurine or methotrexate and history of surgery. Robust outcome ascertainment in administrative data hinges on the diagnostic accuracy of the codes used to define each IMID. For our high-prevalence endpoints, external validation studies demonstrate excellent positive‐predictive values (PPVs): psoriasis (L40*) shows a PPV of 93% in the Danish National Patient Register when ≥ 2 contacts are required, while rheumatoid arthritis (M05*/M06*) achieves PPVs of 88% and sensitivities of 91% in the same registry when linked to the DANBIO clinical database [14, 15]. Multiple sclerosis (G35) algorithms combining one inpatient or two outpatient claims yield PPVs ≥ 90% and sensitivities > 85% across Canadian and U.S. health-system datasets [16]. Although fewer validation papers exist for rarer IMIDs, the available evidence supports a high coding specificity for specialist-managed conditions. Importantly, TriNetX derives diagnoses directly from electronic health-record problem lists, encounter diagnoses and pathology‐confirmed narrative notes; each record must pass proprietary completeness and plausibility checks before network release [17]. These validation data provide confidence that our adjudicated person-time accurately captures true incident IMIDs across the spectrum of interest, strengthening the internal validity of the comparative risk estimates generated in this study.

### Statistical Analysis

All statistical analyses were conducted using the TriNetX software using the browser-based real-time analytics feature, TriNetx Live (TriNetX LLC, Cambridge, MA). Baseline characteristics of cohorts were described using means, standard deviations, and proportions. Covariates based on demographics, comorbid diseases, laboratory parameters and historical IBD medication use were identified. One-to-one (1:1) propensity score matching (PSM) was performed to balance the following covariates between groups: age, gender, race, nicotine dependence, CD location, fistulizing disease, surgery, history of corticosteroid use and immunomodulator use. TriNetX platform utilizes input matrices of the user-identified covariates to conduct logistic regression analysis to obtain propensity scores for all individual subjects. The propensity scores generated are used to match patients using greedy nearest-neighbor algorithms with a caliper width of 0.1 pooled standard deviations. TriNetX randomizes the order of rows to eliminate bias resulting from nearest-neighbor algorithms. Standardized mean difference after PSM indicate the success of matching a covariate between the two cohorts. A standardized mean difference < 0.1 indicates that the difference between the cohorts for the co-variate is small. Time-to-event outcomes were analyzed with Kaplan–Meier curves and compared with log-rank tests; adjusted hazard ratios (aHRs) with 95% confidence intervals (CIs) were obtained from Cox proportional-hazards models that incorporated the matched pairs. The proportional-hazards assumption was verified with Schoenfeld residuals. The numbers are validated by comparing them with output from SAS version 9.4 (SAS Institute, Cary, NC, USA). Missing laboratory data were handled with complete-case analysis because imputation is not currently supported within the TriNetX Live environment.

### Ethical Considerations

The study used only de-identified data certified as such by TriNetX; therefore, institutional review board approval and informed consent were not required under 45 CFR §46.102(f).

## Results

### Cohorts and Baseline Characteristics

A total of 14,360 patients with IBD switched biologic therapy after discontinuing the initial TNFi. Of these, 5962 (41.5%) initiated a second TNFi, and 8398 (58.5%) started ustekinumab or vedolizumab. Second-TNFi cohort was younger (mean age 33.9 ± 16.9 years vs 39.5 ± 17.1 years, *p* < 0.0001) and less often female (49.3% vs 51.3%, *p* = 0.01). The majority of patients in both cohorts had CD (80.8% vs 77.2%). The mean duration from diagnosis to first-line TNFi initiation was 491.1 days. The mean duration of infliximab prior to switching to adalimumab was 592.9 days and the mean duration of adalimumab prior to switching to infliximab was 539.6 days. The mean duration of TNFi prior to switching to ustekinumab or vedolizumab was 984.7 days. The mean follow-up was 1354.1 days in the second-TNFi cohort and 1315.7 days in the reference treatment cohort. Complete details regarding the co-variates before and after PSM can be found in Table 1.

**Table 1:** Baseline characteristics before and after propensity-score matching in patients switching to a Second TNFi cohort versus Ustekinumab/Vedolizumab

Characteristic	Before matching				After matching			
	Second TNFi (* n* = 5962)	Reference treatment cohort (*n* = 8,398)	*P*	SMD	Second TNFi (*n* = 5316)	Reference treatment cohort (*n* = 5316)	*P*	SMD
Age at index, mean ± SD (y)	33.9 ± 16.9	39.5 ± 17.1	0.00	0.33	35.6 ± 16.9	35.9 ± 15.9	0.38	0.02
White	4403 (73.9)	6353 (75.7)	0.014	0.04	3998 (75.2)	3971 (74.7)	0.546	0.01
Female	2938 (49.3)	4305 (51.3)	0.019	0.04	2636 (49.6)	2650 (49.9)	0.786	0.01
Black or African American	676 (11.3)	680 (8.1)	< 0.01	0.11	504 (9.5)	530 (10.0)	0.395	0.02
Hispanic or Latino	325 (5.5)	371 (4.4)	0.004	0.05	268 (5.0)	265 (5.0)	0.894	0.00
Asian	113 (1.9)	194 (2.3)	0.090	0.03	109 (2.0)	103 (1.9)	0.677	0.01
Ulcerative colitis	2,784 (46.7)	4,291 (51.1)	< 0.01	0.08	2,567 (48.8%)	2,567 (48.3%)	0.52	0.01
Crohn’s disease	4818 (80.8)	6484 (77.2)	< 0.01	0.09	4201 (79.0)	4161 (78.3)	0.344	0.02
CD small + large intestine	2552 (42.8)	3875 (46.1)	< 0.01	0.07	2355 (44.3)	2311 (43.5)	0.390	0.02
CD large intestine	2545 (42.7)	3815 (45.4)	0.001	0.06	2335 (43.9)	2280 (42.9)	0.282	0.02
CD small intestine	2218 (37.2)	3498 (41.6)	< 0.01	0.09	2072 (39.0)	2012 (37.9)	0.232	0.02
Nicotine dependence	732 (12.3)	1067 (12.7)	0.446	0.01	670 (12.6)	655 (12.3)	0.660	0.01
Anal fistula	655 (11.0)	875 (10.4)	0.278	0.02	565 (10.6)	570 (10.7)	0.875	0.00
Intestinal fistula	472 (7.9)	664 (7.9)	0.982	0.00	412 (7.8)	402 (7.6)	0.715	0.01
CD w/ fistula	397 (6.7)	717 (8.5)	< 0.01	0.07	387 (7.3)	390 (7.3)	0.911	0.00
CD unspecified fistula	369 (6.2)	582 (6.9)	0.078	0.03	347 (6.5)	342 (6.4)	0.844	0.00
CD large-intestine fistula	363 (6.1)	601 (7.2)	0.012	0.04	345 (6.5)	335 (6.3)	0.692	0.01
Prednisone use	3833 (64.3)	5563 (66.2)	0.015	0.04	3439 (64.7)	3477 (65.4)	0.440	0.01
Methylprednisolone use	2893 (48.5)	3769 (44.9)	< 0.01	0.07	2451 (46.1)	2501 (47.1)	0.331	0.02
Budesonide use	1811 (30.4)	3642 (43.4)	< 0.01	0.27	1801 (33.9)	1794 (33.8)	0.886	0.00
Azathioprine use	1511 (25.3)	2098 (25.0)	0.622	0.01	1331 (25.0)	1349 (25.4)	0.688	0.01
Methotrexate use	825 (13.8)	1291 (15.4)	0.011	0.04	759 (14.3)	756 (14.2)	0.934	0.00
Excision surgery	1 995 (33.5)	2761 (32.9)	0.463	0.01	1719 (32.3)	1764 (33.2)	0.352	0.02
Intestinal resection	460 (7.7)	731 (8.7)	0.034	0.04	421 (7.9)	425 (8.0)	0.886	0.00
Laparoscopic intestinal excision	235 (3.9)	424 (5.1)	0.002	0.05	227 (4.3)	233 (4.4)	0.775	0.01
Partial colectomy	82 (1.4)	123 (1.5)	0.657	0.01	74 (1.4)	68 (1.3)	0.612	0.01
Total abdominal colectomy (no proctectomy)	18 (0.3)	43 (0.5)	0.056	0.03	18 (0.3)	16 (0.3)	0.731	0.01
Total abdominal colectomy with proctectomy	12 (0.2)	21 (0.3)	0.547	0.01	12 (0.2)	15 (0.3)	0.563	0.01

*TNFi* tumour-necrosis-factor inhibitor, *Uste* ustekinumab, *Vedo* vedolizumab, *SD* standard deviation, *SDm* absolute standardised mean difference, *CD* Crohn’s disease, *y* years

### Primary Outcome

**Fig. 1:**
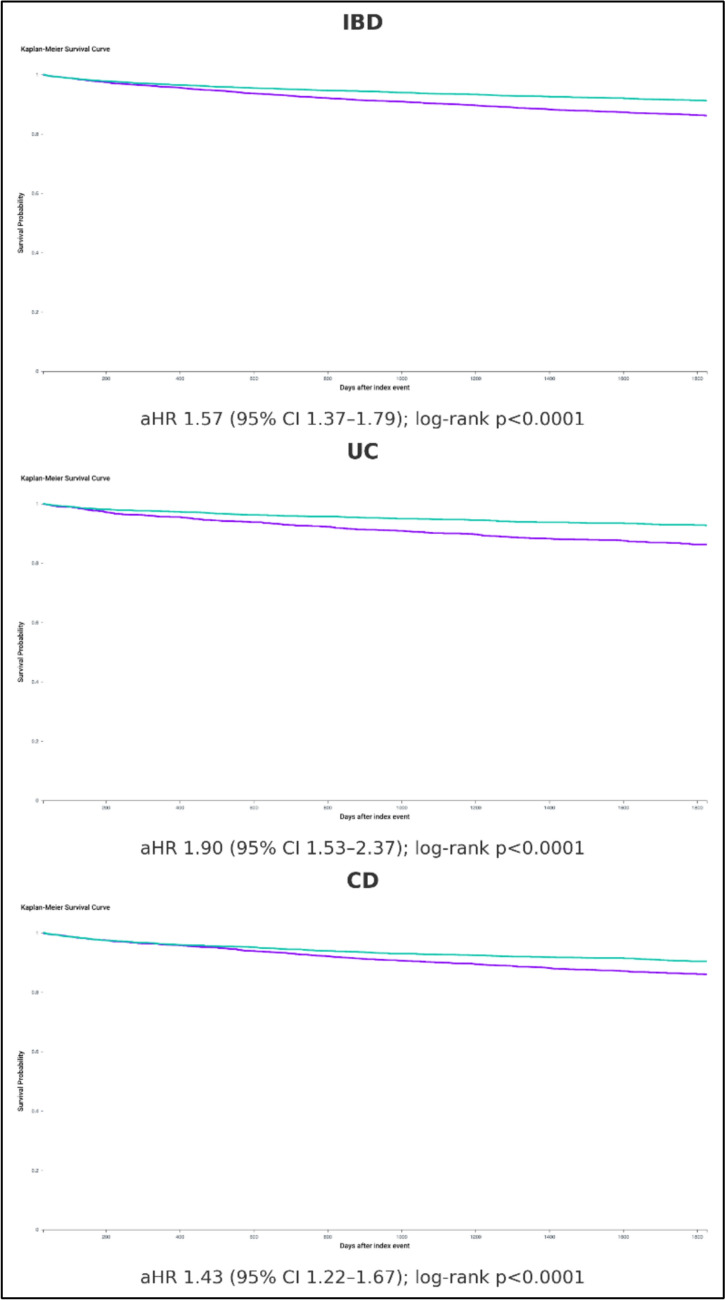
**A** Kaplan–Meier curve for composite IMID-free survival in the overall IBD cohort (Second TNFi vs Ustekinumab/Vedolizumab). **B** Kaplan–Meier curve for composite IMID-free survival in ulcerative colitis (Second TNFi vs Ustekinumab/Vedolizumab). **C** Kaplan–Meier curve for composite IMID-free survival in Crohn’s disease (Second TNFi vs Ustekinumab/Vedolizumab)

At five years, the composite incidence of any new IMID was 10.8% (*n* = 576) in the second-TNFi cohort and 6.9% (*n* = 370) in the reference treatment cohort, corresponding to an aHR of 1.57 (95% CI, 1.37–1.79; log rank *p* < 0.0001) (Table 2). In the UC cohort, composite IMID risk was 10.9% (*n* = 233) with second TNFi and 5.8% (*n* = 125) with the reference treatment cohort (aHR 1.90, 95% CI 1.53–2.37). In the CD cohort, risks were 10.9% (*n* = 385) and 7.7% (*n* = 272), respectively (aHR 1.43, 95% CI 1.22–1.67) (Fig. 1). When psoriasis, RA, and AS were excluded from the composite outcome, five-year incidences were 5.07% (*n* = 257) for second TNFi and 3.04% (*n* = 154) for the reference treatment cohort(aHR 1.67, 95% CI 1.37–2.05). Applying a six-month wash-out to limit protopathic bias yielded composite rates of 8.9% (*n* = 439) versus 5.1% (*n* = 255) and an increased risk of IMID in the second-TNFi cohort (aHR 1.74, 95% CI 1.49–2.03). Comparing the second-TNFi cohort to individual biologic therapies in the reference treatment cohort, we also observed an increased risk of IMID compared to ustekinumab (aHR 1.35, 95% CI 1.16–1.56) and vedolizumab (aHR 1.66, 95% CI 1.40–1.98).

**Table 2:** Five-year risk of new-onset immune-mediated inflammatory diseases after biologic switch in inflammatory bowel disease

IMID outcome	Cohort	Events *N* (%)	aHR^a^	95% CI	*P* value
Composite (all IBD)	Second TNFi	576 (10.8)	**1.57**	1.37–1.79	** < 0.0001**
	Reference treatment cohort	370 (6.9)	Ref	–	**–**
Composite—ulcerative colitis	Second TNFi	233 (10.9)	**1.90**	1.53–2.37	** < 0.0001**
	Reference treatment cohort	125 (5.8)	Ref	–	**–**
Composite—Crohn’s disease	Second TNFi	385 (10.9)	**1.43**	1.22–1.67	** < 0.0001**
	Reference treatment cohort	272 (7.7)	Ref	–	**–**
Composite^b^ (excl. RA/PS/AS)	Second TNFi	257 (5.07)	**1.67**	1.37–2.05	** < 0.0001**
	Reference treatment cohort	154 (3.04)	Ref	–	–
Composite^c^ (6-mo wash-out)	Second TNFi	439 (8.9)	**1.74**	1.49–2.03	** < 0.0001**
	Reference treatment cohort	255 (5.1)	Ref	–	**–**
Psoriasis	Second TNFi	203 (3.8)	**1.66**	1.32–2.08	** < 0.0001**
	Reference treatment cohort	121 (2.2)	Ref	–	–
Ankylosing spondylitis	Second TNFi	30 (0.56)	1.72	0.95–3.13	0.06
	Reference treatment cohort	17 (0.32)	Ref	–	–
Rheumatoid arthritis	Second TNFi	169 (3.1)	**1.74**	1.35–2.24	** < 0.0001**
	Reference treatment cohort	96 (1.8)	Ref	–	–
Systemic lupus erythematosus	Second TNFi	48 (0.90)	**1.57**	1.00–2.49	**0.04**
	Reference treatment cohort	30 (0.56)	Ref	–	–
Vitiligo	Second TNFi	12 (0.22)	1.53	0.62–3.75	0.34
	Reference treatment cohort	< 10	–	–	–
Hidradenitis suppurativa	Second TNFi	83 (1.56)	**1.63**	1.14–2.31	0.005
	Reference treatment cohort	50 (0.94)	Ref	–	–
Polymyalgia rheumatica	Second TNFi	< 10	NA	NA	NA
	Reference treatment cohort	< 10	–	–	–
Sarcoidosis	Second TNFi	< 10	NA	NA	NA
	Reference treatment cohort	< 10	–	–	–
Addison disease	Second TNFi	22 (0.41)	1.66	0.83–3.31	0.14
	Reference treatment cohort	13 (0.24)	Ref	–	–
Graves’ disease	Second TNFi	< 10	0.67	0.29–1.58	0.36
	Reference treatment cohort	13 (0.24)	Ref	–	–
Autoimmune hepatitis	Second TNFi	20 (0.37)	**4.02**	1.51–10.72	**0.002**
	Reference treatment cohort	10 (0.18)	Ref	–	–
Primary biliary cholangitis	Second TNFi	10 (0.18)	0.65	0.26–1.59	0.34
	Reference treatment cohort	12 (0.22)	Ref	–	–
Pernicious anemia	Second TNFi	14 (0.26)	2.27	0.87–5.91	0.08
	Reference treatment cohort	10 (0.18)	Ref	–	–
Multiple sclerosis	Second TNFi	< 10	NA	NA	NA
	Reference treatment cohort	< 10	–	–	–
Myasthenia gravis	Second TNFi	< 10	NA	NA	NA
	Reference treatment cohort	0	–	–	–
Immune thrombocytopenic purpura	Second TNFi	< 10	NA	NA	NA
	Reference treatment cohort	< 10	–	–	–

Bold values indicate statistically significant results

*IMID* immune-mediated inflammatory disease, *TNFi* tumour-necrosis-factor inhibitor, *Uste* ustekinumab, *Vedo* vedolizumab, *aHR* adjusted hazard ratio, *CI* confidence interval, *Ref* reference group

*NA* = not applicable because event numbers were < 10 in both cohorts

^a^*aHR* adjusted hazard ratio, *Ref* Reference treatment cohort cohort is the reference

^b^Composite excluding rheumatoid arthritis (RA), psoriasis (PS) and ankylosing spondylitis (AS)

^c^Composite analysis that excludes IMID events occurring within the first six months after the biologic switch to minimize protopathic bias

### Secondary Outcomes

For individual IMIDs, five-year risks after second TNFi versus the reference treatment cohort were: psoriasis 3.8% vs 2.2%, aHR 1.66 (95% CI 1.32–2.08; *p* < 0.0001); AS 0.56% vs 0.32%, aHR 1.72 (95% CI 0.95–3.13; *p* = 0.06); RA 3.1% vs 1.8%, aHR 1.74 (95% CI 1.35–2.24; *p* < 0.0001); SLE 0.90% vs 0.56%, aHR 1.57 (95% CI 1.00–2.49; *p* = 0.04); HS 1.56% vs 0.94%, aHR 1.63 (95% CI 1.14–2.31; *p* = 0.005). AIH occurred in 0.37 vs 0.18% with an aHR of 4.02 (95% CI 1.51–10.72; *p* = 0.002). Addison disease, primary biliary cholangitis (PBC), pernicious anemia, multiple sclerosis, myasthenia gravis, sarcoidosis, polymyalgia rheumatica, and immune thrombocytopenic purpura each had fewer than 25 events (Table 2).

Subgroup analysis by age revealed composite IMID incidences of 9.5% (*n* = 220) with second TNFi and 5.5% (*n* = 128) with the reference treatment cohort among patients aged 18–39 years (aHR 1.77, 95% CI 1.43–2.21). In those ≥ 40 years old, incidences were 11.5% (*n* = 258) vs 7.6% (*n* = 171) (aHR 1.53, 95% CI 1.26–1.85). By sex, men showed rates of 8.9% (*n* = 163) vs 5.0% (*n* = 91) (aHR 1.84, 95% CI 1.42–2.38), and women showed 12.9% (*n* = 308) vs 8.4% (*n* = 201) (aHR 1.55, 95% CI 1.30–1.85) (Table 3).

**Table 3:** Subgroup analysis of five-year composite IMID risk after second TNFi versus Reference treatment cohort

Subgroup	Cohort	Events *N* (%)	aHR	95% CI	*P* value
Age 18–39 y	Second TNFi	220 (9.5)	**1.77**	1.43–2.21	** < 0.0001**
	Reference treatment cohort	128 (5.5)	Ref	–	–
Age ≥ 40 y	Second TNFi	258 (11.5)	**1.53**	1.26–1.85	** < 0.0001**
	Reference treatment cohort	171 (7.6)	Ref	–	**–**
Male	Second TNFi	163 (8.9)	**1.84**	1.42–2.38	** < 0.0001**
	Reference treatment cohort	91 (5.0)	Ref	–	–
Female	Second TNFi	308 (12.9)	**1.55**	1.30–1.85	** < 0.0001**
	Reference treatment cohort	201 (8.4)	Ref	–	–

Bold values indicate statistically significant results

*IMID* immune-mediated inflammatory disease, *TNFi* tumour-necrosis-factor inhibitor, *Uste* ustekinumab, *Vedo* vedolizumab, *aHR* adjusted hazard ratio, *CI* confidence interval, *Ref* reference group, *y* years

### Risk Factors for IMID in Second-TNFi Cohort

Within the second-TNFi cohort, age 40–64 years (HR 1.36, 95% CI 1.11–1.67) and age ≥ 65 years (HR 1.35, 95% CI 1.02–1.78) were associated with an increased risk of IMID. Primary sclerosing cholangitis was associated with a higher risk (HR 3.49, 95% CI 2.12–5.74). Methotrexate use in the year before the switch carried an elevated risk (HR 1.35, 95% CI 1.15–1.59), whereas concomitant azathioprine was associated with a lower risk (HR 0.85, 95% CI 0.74–0.99). Male sex had a protective association (HR 0.65, 95% CI 0.57–0.74). Race/ethnicity, nicotine dependence, prior bowel surgery, and recent corticosteroid exposure showed no significant associations with the risk of IMID (Table 4).

**Table 4:** Independent predictors of new-onset IMID in patients receiving a second TNFi

Variable	HR	95% CI
Age 40–64 y	**1.36**	1.11–1.67
Age > 65 y	**1.35**	1.02–1.78
Male sex	**0.65**	0.57–0.74
Primary sclerosing cholangitis	**3.49**	2.12–5.74
Azathioprine co-therapy	**0.85**	0.74–0.99
Methotrexate co-therapy	**1.35**	1.15–1.59
Oral steroids (< 1 y)	1.10	0.96–1.26
IV steroids (< 1 y)	1.05	0.91–1.22
Nicotine dependence	0.96	0.76–1.22
Inpatient surgery history	1.01	0.87–1.15
Outpatient enterectomy	0.77	0.49–1.21
Outpatient colectomy	1.21	0.89–1.63

Bold values indicate statistically significant results

*IMID* immune-mediated inflammatory disease, *TNFi* tumor-necrosis-factor inhibitor, *Uste* ustekinumab, *Vedo* vedolizumab, *aHR* adjusted hazard ratio, *CI* confidence interval, *Ref* reference group, *y* years, *HR* hazard ratio

## Discussion

In this large real-world cohort, switching within class to a second TNFi was associated with a 57% higher adjusted hazard of developing a new IMID over five years compared to switching to ustekinumab or vedolizumab. The excess risk was consistent across UC and CD and persisted after accounting for early detection bias and after excluding psoriasis, RA, and AS. Psoriasis and RA were the most frequent events. Older age, methotrexate use, and PSC were associated with increased risk of IMID, whereas male sex and previous azathioprine use was associated with a lower risk in the second-TNFi cohort.

The magnitude of the composite risk after the second TNFi aligns closely with nationwide Danish–French data in which the incidence of psoriasis, RA and hidradenitis reached 5.3 per 1000 person-years under first-line TNFi compared with 3.0 per 1000 person-years in biologic-naïve controls (pooled HR 1.76) [10]. Converting our five-year cumulative incidence (10.8%) to person-time (~ 30 000 PY) yields 36 events per 1000 PY, reflecting the enrichment that occurs when only patients who have already failed one anti-TNFi are analyzed. Smaller single-center series have reported paradoxical IMID rates between 4 and 10% after a first TNFi [18, 19]; our data after a second exposure therefore suggest a stepwise accumulation of risk. In contrast, the 6.9% five-year incidence after ustekinumab or vedolizumab is comparable to the 5-year 6.1% reported in a French administrative comparison of 10,712 s-TNFi and 3282 ustekinumab initiators (HR 0.63 vs TNFi) [13]. Our aHR of 0.64 for ustekinumab/vedolizumab relative to second TNFi therefore reproduces that observation in an independent data set, adds vedolizumab to the comparison, and incorporates time-to-event modelling with a three-fold longer run-in. Methodological differences among studies help explain residual discrepancies. Ward et al. matched first-line TNFi users to unexposed patients and applied a six-month lag to mitigate protopathic bias; our primary analysis lacked a lag but the sensitivity wash-out produced essentially identical estimates, arguing against reverse causation [10].

Biologic mechanism offers a biologically plausible basis for the differential risks observed. Chronic TNF neutralization skews cytokine signaling toward type I interferon, interleukin-17 and interleukin-23 pathways, promotes plasmacytoid dendritic-cell activation and breaks B-cell tolerance, facilitating neo-autoantibody formation [9]. Up-regulation of interferon-stimulated genes and dermal IL-17 has been demonstrated in lesional skin from anti-TNFi-induced psoriasis [20]. Genetic predisposition modulates this effect: HLA-C*06:02 carriers have higher odds of paradoxical psoriasis, and variants in TYK2 and IL23R are shared risk alleles for both IBD and psoriasis [21, 22]. In contrast, ustekinumab directly inhibits IL-12/23 signaling and vedolizumab confines lymphocyte blockade to the gut; neither perturbs systemic TNF [23, 24]. Observational signals of lower IMID incidence, specifically concentrated to case series and case reports, with these agents therefore accord with mechanism, and our data extend those signals beyond psoriasis to RA, HS and autoimmune hepatitis. Notably, HS risk fell from 1.56% after second TNFi to 0.94% after ustekinumab or vedolizumab, echoing Licata et al., who found scarce reports of HS under vedolizumab while reporting their case report [25].

Baseline IBD severity and prior immunomodulator exposure can confound safety estimates. We adjusted for corticosteroids, bowel resections and hospitalizations, variables validated as surrogates for disease activity [26, 27]. The protective effect of concomitant azathioprine we observed (HR 0.85) mirrors data from Soh et al., who reported reduced cumulative incidence of adverse skin lesions when azathioprine was combined with infliximab (aHR 0.72); by dampening anti-drug antibodies, thiopurines may prevent immune complex formation and complement activation, proposed triggers of paradoxical autoimmunity [28]. Conversely, methotrexate exposure was associated with higher IMID risk (HR 1.35), consistent with hypotheses that weekly spikes in circulating TNF render blockade incomplete, sustaining autoreactive T-cell pools [6]. Age-related immune senescence may explain the stepwise rise in risk across decades, while the lower risk in male sex aligns with stronger type I interferon responses in women [29, 30].

Our absolute psoriasis incidence (3.8% second TNFi; 2.2% Reference treatment cohort) is lower than the 6.0% pooled rate in a meta-analysis of patients with IBD on TNFi by Xie et. al but approaches the 3% cumulative rate in an observational cohort study by Bae et al. [11, 31]. The higher baseline exposure to infliximab in Xie et al’s study compared with predominantly adalimumab in ours may account for differences, as infliximab has been most consistently linked to paradoxical skin disease [32]. For RA, our 3.1% five-year rate after a second TNFi exceeds the cumulative 1.76% reported by Ward et al. under initial TNFi; the step-up could reflect unmeasured genetic predisposition in patients losing response to the first agent [10]. Early reports in AS described new-onset CD or UC during TNFi therapy, establishing a “reverse paradox” from joint to gut [20]. Our switch cohort demonstrates the converse: IBD patients who remained on TNFi developed AS almost twice as often [aHR for AS (1.72)] as those who moved to ustekinumab or vedolizumab, a pattern echoed by Subramaniam et al., who found incident spondyloarthropathy in 6% of 149 TNFi-treated IBD patients (4.6/100 patient-years) despite good intestinal control [33]. Mechanistically, sustained TNF blockade down-regulates membrane-bound TNF on macrophages, curtailing Fas-mediated deletion of autoreactive T cells and amplifying IL-17/23 and type-I-interferon circuits that drive entheseal inflammation and psoriasiform change [34]. Because ustekinumab directly targets IL-12/23 and vedolizumab confines lymphocyte blockade to the gut, neither perturbs systemic TNF; pooled safety data (> 12,000 ustekinumab- and > 4000 vedolizumab-exposed patient-years) reveals no excess de-novo IBD or axial spondylarthritis, reinforcing their favorable extra-intestinal profile [35, 36]. Collectively, these findings support vigilant bidirectional monitoring when TNFi are re-used and favors class switching when clinically feasible. We noted no signal for multiple sclerosis despite prior reports linking infliximab to demyelination; only eight events occurred, all censoring before exposure to a second agent, highlighting the rarity of this complication in modern practice [37]. Autoimmune hepatitis, however, was fourfold more common after second TNFi; anti-nuclear and anti-smooth-muscle antibody induction under TNFi has been documented, and our findings match isolated case series, underscoring the need for liver enzyme monitoring after class re-challenge [38–40].

There are several implications based on our findings. First, when loss of response to a first TNFi necessitates escalation, class switching appears to confer a clinically meaningful 3.9% absolute risk reduction in incident IMID over five years. Second, older patients, women and those with primary sclerosing cholangitis represent subgroups in whom class switching may be particularly advantageous. Third, concomitant azathioprine may mitigate IMID risk, supporting its short-term use during TNFi induction, whereas methotrexate appears neutral or harmful in this context. Finally, systematic pharmacovigilance using harmonized IMID definitions would refine risk estimates for emerging agents such as janus-kinase inhibitors, sphingosine-1-phosphate modulators and selective IL-23 antagonists that are rapidly entering clinical practice [41].

Strengths of our investigation include sample size and separate evaluation of UC and CD, which displayed parallel risk patterns. Methodologically, our study leverages a large, racially diverse database, long follow-up and robust propensity-score adjustment including calendar year, immunomodulator exposure and steroid use. We present sensitivity analyses with a six-month lag that yielded consistent results, strengthening causal inference. We also analyzed different co-variates within the second-TNFi cohort which may impact the incidence of IMIDs.

Our study also has several limitations that warrant consideration. The indication for biologic change could not be determined from the database. It is possible a higher proportion of patients in the second TNFi cohort developed secondary loss of response or side effects to the initial TNFi due to anti-drug antibodies. It is unclear if this impacted the development of de-novo IMIDs in this cohort. Since exact discontinuation and switching dates are not consistently available in the source EHR data, we could not reliably construct on-treatment risk windows; our primary analysis was intention-to-treat. This choice may attenuate differences between cohorts but aligns with our goal of estimating the association of treatment initiation with incident IMIDs irrespective of subsequent treatment duration or changes. Our composite outcome combined disparate pathologies; however, stratified analyses reproduced the main effect across dominant IMID phenotypes. Limitations comprise reliance on coded diagnoses, potential channeling bias whereby dermatologic history influenced biologic choice, and lack of serum cytokine or genetic data to explore mechanistic correlates. Future prospective registries incorporating biospecimens could test whether HLA-C*06:02 or interferon-signature scores predict paradoxical IMID and might guide preventative strategies such as low-dose methotrexate or early class-switching. Extended follow-up beyond five years is also warranted to determine whether divergence in IMID risk persists, narrows or widens with prolonged exposure.

In summary, within-class switching to a second TNFi was associated with higher five-year rates of composite and individual IMIDs than class switching to ustekinumab or vedolizumab in a large real-world IBD cohort. These findings corroborate and extend prior population-based studies, quantify absolute risk differences, and highlight patient-level modifiers that may aid personalized biologic sequencing. Continued surveillance and mechanistic research are needed to refine risk stratification and optimize long-term safety for patients requiring successive biologic therapies.

## Supplementary Information

Below is the link to the electronic supplementary material.

Supplementary file1 (DOCX 22 KB)

## Data Availability

The data underlying this article will be shared at a reasonable request by the corresponding author.

## Abbreviations

aHR: Adjusted hazard ratio
AIH: Autoimmune hepatitis
AS: Ankylosing spondylitis
CD: Crohn’s disease
CI: Confidence interval
HS: Hidradenitis suppurativa
HR: Hazard ratio
IBD: Inflammatory bowel disease
IMID: Immune-mediated inflammatory disease
ITP: Immune thrombocytopenic purpura
PBC: Primary biliary cholangitis
PSC: Primary sclerosing cholangitis
PY: Person-years
RA: Rheumatoid arthritis
SLE: Systemic lupus erythematosus
TNFi: Tumor necrosis factor inhibitor
UC: Ulcerative colitis
Uste: Ustekinumab
Vedo: Vedolizumab
