# Supplementary material for: Risk of Incident Immune-Mediated Inflammatory Diseases with Second Tumor Necrosis Factor Inhibitor Versus Alternative Biologic Therapy in Patients with Inflammatory Bowel Disease and First TNFi Exposure: A Real-World Cohort Study
Source: Dig Dis Sci. 2025 Dec 9;71(4):1221–31. doi: 10.1007/s10620-025-09598-4 (PMC13144199; doi:10.1007/s10620-025-09598-4)
Supplement: Supplementary file 1 — Supplementary file1 (DOCX 22 KB) [file 10620_2025_9598_MOESM1_ESM.docx]

**Table 1:**
Baseline Characteristics Before and After Propensity-Score Matching in Patients Switching to a Second TNFi versus Ustekinumab/Vedolizumab

| Characteristic | Before matching | | | | After matching | | | |
| --- | --- | --- | --- | --- | --- | --- | --- | --- |
|  | **Second TNFi N (%)** | **Uste/Vedo N (%)** | **p** | **SDm** | **Second TNFi N (%)** | **Uste/Vedo N (%)** | **p** | **SDm** |
| Age at index, mean ± SD (y) | 33.9 ± 16.9 | 39.5 ± 17.1 | 0.00 | 0.33 | 35.6 ± 16.9 | 35.9 ± 15.9 | 0.38 | 0.02 |
| White | 4 403 (73.9) | 6 353 (75.7) | 0.014 | 0.04 | 3 998 (75.2) | 3 971 (74.7) | 0.546 | 0.01 |
| Female | 2 938 (49.3) | 4 305 (51.3) | 0.019 | 0.04 | 2 636 (49.6) | 2 650 (49.9) | 0.786 | 0.01 |
| Black or African-American | 676 (11.3) | 680 (8.1) | <0.001 | 0.11 | 504 (9.5) | 530 (10.0) | 0.395 | 0.02 |
| Hispanic or Latino | 325 (5.5) | 371 (4.4) | 0.004 | 0.05 | 268 (5.0) | 265 (5.0) | 0.894 | 0.00 |
| Asian | 113 (1.9) | 194 (2.3) | 0.090 | 0.03 | 109 (2.0) | 103 (1.9) | 0.677 | 0.01 |
| Crohn’s disease (K50) | 4 818 (80.8) | 6 484 (77.2) | <0.001 | 0.09 | 4 201 (79.0) | 4 161 (78.3) | 0.344 | 0.02 |
| CD small + large intestine (K50.8) | 2 552 (42.8) | 3 875 (46.1) | <0.001 | 0.07 | 2 355 (44.3) | 2 311 (43.5) | 0.390 | 0.02 |
| CD large intestine (K50.1) | 2 545 (42.7) | 3 815 (45.4) | 0.001 | 0.06 | 2 335 (43.9) | 2 280 (42.9) | 0.282 | 0.02 |
| CD small intestine (K50.0) | 2 218 (37.2) | 3 498 (41.6) | <0.001 | 0.09 | 2 072 (39.0) | 2 012 (37.9) | 0.232 | 0.02 |
| Nicotine dependence | 732 (12.3) | 1 067 (12.7) | 0.446 | 0.01 | 670 (12.6) | 655 (12.3) | 0.660 | 0.01 |
| Anal fistula | 655 (11.0) | 875 (10.4) | 0.278 | 0.02 | 565 (10.6) | 570 (10.7) | 0.875 | 0.00 |
| Intestinal fistula | 472 (7.9) | 664 (7.9) | 0.982 | 0.00 | 412 (7.8) | 402 (7.6) | 0.715 | 0.01 |
| CD w/ fistula (K50.813) | 397 (6.7) | 717 (8.5) | <0.001 | 0.07 | 387 (7.3) | 390 (7.3) | 0.911 | 0.00 |
| CD unspecified fistula (K50.913) | 369 (6.2) | 582 (6.9) | 0.078 | 0.03 | 347 (6.5) | 342 (6.4) | 0.844 | 0.00 |
| CD large-intestine fistula (K50.113) | 363 (6.1) | 601 (7.2) | 0.012 | 0.04 | 345 (6.5) | 335 (6.3) | 0.692 | 0.01 |
| Prednisone use | 3 833 (64.3) | 5 563 (66.2) | 0.015 | 0.04 | 3 439 (64.7) | 3 477 (65.4) | 0.440 | 0.01 |
| Methylprednisolone use | 2 893 (48.5) | 3 769 (44.9) | <0.001 | 0.07 | 2 451 (46.1) | 2 501 (47.1) | 0.331 | 0.02 |
| Budesonide use | 1 811 (30.4) | 3 642 (43.4) | <0.001 | 0.27 | 1 801 (33.9) | 1 794 (33.8) | 0.886 | 0.00 |
| Azathioprine use | 1 511 (25.3) | 2 098 (25.0) | 0.622 | 0.01 | 1 331 (25.0) | 1 349 (25.4) | 0.688 | 0.01 |
| Methotrexate use | 825 (13.8) | 1 291 (15.4) | 0.011 | 0.04 | 759 (14.3) | 756 (14.2) | 0.934 | 0.00 |
| Excision surgery | 1 995 (33.5) | 2 761 (32.9) | 0.463 | 0.01 | 1 719 (32.3) | 1 764 (33.2) | 0.352 | 0.02 |
| Intestinal resection | 460 (7.7) | 731 (8.7) | 0.034 | 0.04 | 421 (7.9) | 425 (8.0) | 0.886 | 0.00 |
| Laparoscopic intestinal excision | 235 (3.9) | 424 (5.1) | 0.002 | 0.05 | 227 (4.3) | 233 (4.4) | 0.775 | 0.01 |
| Partial colectomy | 82 (1.4) | 123 (1.5) | 0.657 | 0.01 | 74 (1.4) | 68 (1.3) | 0.612 | 0.01 |
| Total abdominal colectomy (no proctectomy) | 18 (0.3) | 43 (0.5) | 0.056 | 0.03 | 18 (0.3) | 16 (0.3) | 0.731 | 0.01 |
| Total abdominal colectomy with proctectomy | 12 (0.2) | 21 (0.3) | 0.547 | 0.01 | 12 (0.2) | 15 (0.3) | 0.563 | 0.01 |

*TNFi = tumour-necrosis-factor inhibitor; Uste = ustekinumab; Vedo = vedolizumab; SD = standard deviation; SDm = absolute standardised mean difference; CD = Crohn’s disease; y = years.*

ICD codes:

| **Diagnosis** | **Code** |
| --- | --- |
| Crohn’s disease [regional enteritis] | K50 |
| Ulcerative colitis | K51 |
| Ankylosing spondylitis | M45 |
| Rheumatoid arthritis with rheumatoid factor | M05 |
| Other rheumatoid arthritis | M06 |
| Psoriasis | L40 |
| Polymyalgia rheumatica | M35.3 |
| Vitiligo | L80 |
| Hidradenitis suppurativa | L73.2 |
| Sarcoidosis | D86 |
| Sjögren syndrome | M35.0 |
| Systemic lupus erythematosus (SLE) | M32 |
| Lupus erythematosus | L93 |
| Primary adrenocortical insufficiency (Addison’s) | E27.1 |
| Thyrotoxicosis with diffuse goiter (Graves) | E05.0 |
| Autoimmune thyroiditis | E06.3 |
| Primary biliary cirrhosis | K74.3 |
| Autoimmune hepatitis | K75.4 |
| Vitamin B12-deficiency anemia (due to intrinsic-factor deficiency) | D51.0 |
| Multiple sclerosis | G35 |
| Myasthenia gravis | G70.0 |
| Immune thrombocytopenic purpura | D69.3 |
| Systemic sclerosis [scleroderma] | M34 |

| **Medication (biologic / small-molecule)** | **RxNorm code** |
| --- | --- |
| infliximab | 191 831 |
| adalimumab | 327 361 |
| vedolizumab | 1538 097 |
| ustekinumab | 847 083 |
| golimumab | 819 300 |
| risankizumab | 2166 040 |
| tofacitinib | 1357 536 |
| upadacitinib | 2196 092 |
